# A Blueprint for Full Collective Flood Risk Estimation: Demonstration for European River Flooding

**DOI:** 10.1111/risa.12747

**Published:** 2016-12-29

**Authors:** Francesco Serinaldi, Chris G. Kilsby

**Affiliations:** ^1^ School of Civil Engineering and Geosciences Newcastle University Newcastle Upon Tyne UK; ^2^ Willis Research Network London UK

**Keywords:** Collective flood risk, dynamic copula space‐time modeling, European rivers

## Abstract

Floods are a natural hazard evolving in space and time according to meteorological and river basin dynamics, so that a single flood event can affect different regions over the event duration. This physical mechanism introduces spatio‐temporal relationships between flood records and losses at different locations over a given time window that should be taken into account for an effective assessment of the collective flood risk. However, since extreme floods are rare events, the limited number of historical records usually prevents a reliable frequency analysis. To overcome this limit, we move from the analysis of extreme events to the modeling of continuous stream flow records preserving spatio‐temporal correlation structures of the entire process, and making a more efficient use of the information provided by continuous flow records. The approach is based on the dynamic copula framework, which allows for splitting the modeling of spatio‐temporal properties by coupling suitable time series models accounting for temporal dynamics, and multivariate distributions describing spatial dependence. The model is applied to 490 stream flow sequences recorded across 10 of the largest river basins in central and eastern Europe (Danube, Rhine, Elbe, Oder, Waser, Meuse, Rhone, Seine, Loire, and Garonne). Using available proxy data to quantify local flood exposure and vulnerability, we show that the temporal dependence exerts a key role in reproducing interannual persistence, and thus magnitude and frequency of annual proxy flood losses aggregated at a basin‐wide scale, while copulas allow the preservation of the spatial dependence of losses at weekly and annual time scales.

## 1. INTRODUCTION

Global costs from weather‐related hazards have increased in recent decades, and losses from storms and floods around the world account for most of the increase.^1^ These loss trends can largely be explained by regional socioeconomic factors such as increasing wealth, population growth, and increasing development in vulnerable areas.^1^, ^2^, ^3^, ^4^ Focusing on flood hazard, the evolution of vulnerability and exposure along with hazard frequency and magnitude related to climate fluctuations require flood risk management strategies to evolve to meet the changing circumstances.^5^, ^6^, ^7^, ^8^ Such policies also imply a shift toward more integrated flood risk management strategies comprising portfolios of structural flood protection assets such as dikes, levees, resilience‐improved residences, and upstream retention areas,^9^ and nonstructural solutions such as property‐level protection, land‐use planning, and insurance arrangements.^3^, ^8^, ^10^ An effective implementation of evolving management strategies must account for key flood characteristics such as their inherent spread over many administrative/physical regions,^11^, ^12^ causing simultaneous collective losses, and their temporal clustering, resulting in flood‐rich and flood‐poor periods.^13^, ^14^, ^15^, ^16^ In particular, modeling flood clustering is paramount to set up mitigation strategies that are not affected by the flood risk perception related to the alternation of flood‐rich and flood‐poor periods. Indeed, experience with floods is often considered to have a great impact on the recognition of risk, resulting, for instance, in a positive relationship between individual flood risk perceptions and demand for flood insurance.^17^, ^18^ Moreover, both the severity of the experienced negative consequences and the timing of the previous experience play an important role, as it can be expected that floods experienced in the distant past have only a limited influence on individual risk perceptions and mitigation behavior later.^18^ In this respect, the International Commission for the Protection of the Rhine (ICPR) estimated that flood awareness mostly diminishes within seven years after a flood and that only catastrophic disasters are remembered in the long term,^19^ and that flood damage is significantly lower in areas where people have recently experienced a flood event, which is attributed to a better preparedness of the population in the direct aftermath of a flood.^20^, ^21^, ^22^

Whether we focus on hazard (water discharge/level magnitude and frequency) or on impacts (losses magnitude and frequency), defining the frequency of regional flood events requires the use of multivariate frequency analysis of discharge values at multiple locations^23^ (e.g., stream gauge stations or grid points of the spatial domain affected by flood events). Different techniques have been proposed in the literature to perform multivariate frequency analysis. Focusing on flood hazard, Student *t* and skew‐*t* copulas were used to model multisite flood events selected by preliminary univariate analyses of annual maximum discharge values and peaks over a fixed percentage threshold.^24^, ^25^ Critical multisite events to be included in the analysis are instead automatically selected by the Heffernan–Tawn conditional model^26^ devised for conditional peaks over threshold.^27^, ^28^, ^29^ Focusing on flood impacts, simplified versions of vine copulas were applied to model the hierarchical spatial dependence structure of flood losses.^30^, ^31^ In these cases, copulas were fitted to monthly peak discharge values yielded by a hydrological model, thus not requiring a preliminary flood event selection.^32^

Irrespective of the method of selecting regional flood events, the modeling strategies mentioned above deal with a limited number of extreme events, generally a few events per year, which are treated as temporally independent. The corresponding simulation procedures also generate temporally independent (but spatially dependent) realizations using, for instance, a Poisson distribution to model the number of events per year.^28^ However, stream flow dynamics are characterized by short‐ and long‐term temporal dependence,^33^, ^34^ which influences not only the interannual fluctuations of the average process but also the magnitude and interarrival times of extreme events,^35^ resulting in extreme values more intense than expected under weak or no temporal dependence, and temporal clustering/overdispersion.^16^ Moreover, since rivers are characterized by different flow regimes based on the dominant climatological drivers in a given region, spatial patterns of potential flood events change along the calendar year according to the seasonal probability to observe simultaneous extreme events over different regions. For practical purposes, such as reinsurance pricing procedures, it is therefore important to know the calendar time of potential losses.

To accomplish this task, we move from event‐based methods to a continuous approach that allows for modeling and simulation of spatially and temporally correlated hazard scenarios at a weekly time scale. The methodology falls in the framework of dynamic conditional copula,^36^, ^37^, ^38^, ^39^ which consists of two modeling stages: first, at‐site (univariate) stream flow properties, including marginal distributions, seasonality, and temporal dependence, are modeled by suitable time series models; since the model residuals (also known as innovations) are temporally independent and identically distributed but preserve spatial correlation, the spatial dependence structure is modeled by a suitable copula‐based multivariate distribution in the second stage. As described in more detail in the next sections, combining at‐site models and the joint distribution of their residuals provides a full spatio‐temporal model suitable for continuous simulation. The copula model is called “dynamic conditional” because the joint distribution of residuals is conditioned on covariates (here, at‐site stream flow dynamics), and can evolve in time accounting for seasonal fluctuations or other dynamics of the spatial correlation structure. The model is applied to 490 stream flow time series recorded across 10 of the largest basins across central and eastern Europe, and its performance is assessed by a set of collective risk indices summarizing “proxy” losses at different spatial and temporal aggregation scales. The article is organized as follows. Section 2. introduces the modeling framework and the data required for parameter estimation and validation. Section 3. discusses empirical results referring to European stream flow data. Finally, conclusions are drawn in Section 4..

## 2. DATA AND METHODOLOGY

### 2.1. Preliminary Remarks on Modeling Strategy

Unlike univariate frequency analysis, where critical events are uniquely defined when the variable of interest exceeds a given critical value, in conventional multivariate flood frequency analysis such a definition is not unique because multiple variables can be combined in different ways according to the nature of the physical process under study and/or the aim of the analysis.^25^, ^40^, ^41^ Dealing with floods over large areas, events usually propagate along the river network spanning up to several days based on geophysical aspects such as time of concentration, antecedent soil moisture conditions, and nature of the meteorological forcings. Event definition and corresponding time scales also depend on specific applications. For example, (re)insurance policies introduce a time window, the so‐called hours clause, that identifies a single event so that the damages occurring in such time interval are ascribed to the same event, thus resulting in a single aggregate loss.^25^ Typically, the hours clause is 72 or 168 hours (one week).

In a classical frequency analysis approach, independent multisite flood events can be identified by sliding widows of size equal to the hours clause, thus selecting the maximum value for each site within the time window. The window is then shifted by the expected flood travel time in the drainage network to guarantee the independence of the events. Finally, the most intense events are selected using severity indices that summarize the overall intensity by the weighted sums of the at‐site return periods (annual exceedance probabilities) of the event's flow values at each location.^25^

Moving from frequency analysis of independent events to continuous modeling, we need a different selection method. It consists of selecting the maximum value of discharge within each calendar week. Even though physical events are not bounded by calendar weeks, this method is reasonable since (i) it yields a large set of possible scenarios within the hours clause; (ii) the use of calendar weeks allows for a detailed analysis of the spatial and temporal evolution of the flood risk through the year, thus providing refined information on how the potential losses aggregate in space and time; and (iii) weekly maxima are a reasonable compromise between threshold‐based selection and the direct use of all daily data; indeed, they allow us to reduce redundant information (and reduce the computational effort) but retain more data than usually used in classical frequency analysis (thus improving the reliability of results).

Since this study focuses on modeling and simulation of spatially and temporally coherent virtual flood hazard scenarios to be used to assess potential losses, we are not interested in the absolute values of the discharge but in their probability of nonexceedance, which describes the degree of rarity of the underlying discharge value. Actually, this is the information required to compute the risk *R*, defined as the product of the hazard *H* and its consequences *D*:^42^, ^43^

(1) R=H·E·V=H·D,

where *E* denotes the exposure (value/humans that are present at the location involved), and *V* is the vulnerability (extent of the exposed values that can be affected by the hazard). To assess the reliability of the modeling approach, Equation (1) is used to compute the basin‐wide aggregated risk (potential losses). Population density and land cover data are used as proxies of exposure *E*, and gross domestic product (GDP) per capita as a proxy of vulnerability *V*.^43^, ^44^ Indeed, the number of people in an area at risk is a basic indicator of flood exposure, while land cover is an indicator of the economic damage resulting from a potential flood. Vulnerability is the most complex element of *R* as it changes in space and time. Given the difficulty of obtaining reliable estimates of *V* on a large scale, GPD per capita is deemed the only proxy available for the entire European Union.^43^ In particular, we assume a (re)insurance point of view indicating the richest areas as the most vulnerable.^44^ As *D* depends on flood magnitude itself according to the so‐called damage curves,^42^ the above assumptions are a simplification; however, they are sufficient in the present context where Equation (1) is simply used to weight *H* at each location in order to define an index of areal hazard summarizing the event intensity at the basin scale.

### 2.2. Data Set

**Figure 1:**
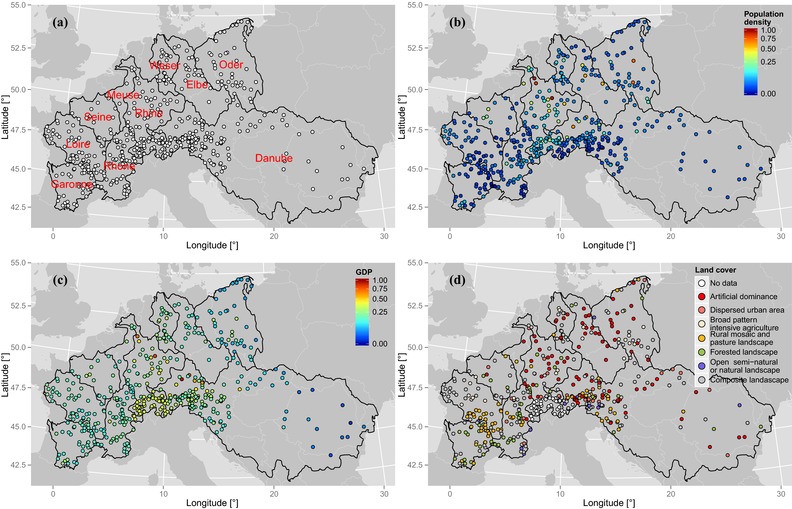
Study area and stream gauge locations (a). Standardized population density (b), GDP (c), and land cover (d).

The data set comprises 490 daily stream flow time series across 10 of the major basins in Europe (data were obtained from the Global Runoff Data Centre, Federal Institute of Hydrology, Koblenz, Germany). The time series were selected in order to have the maximum number of sequences with maximum temporal overlap (namely, 36 years from 1969 to 2005) and less than 10% of missing values; Fig. 1(a) shows the study area and stream gauge locations.

Population density^1^ and GDP per capita^2^ at the NUTS 3 level^3^ referring to 2011 are obtained from the EUROSTAT website,^4^ while land cover (dominant land cover types) has been retrieved from CORINE 2000.^5^ Since *E* and *V* act as weights for *H*, all variables are standardized in the range [0, 1], introducing a saturation at 5% for population density and GDP per capita to avoid boundary effects of extreme low and high values (Figs. 1(b) and 1(c)).^43^ CORINE land cover data consist of eight classes of dominant land cover types, which are also standardized in the unit interval [0, 1] (Fig. 1(d)).

### 2.3. Spatio‐Temporal Model

The methodology proposed in this study aims to model and simulate weekly discharge maxima preserving the spatial and temporal dependence structures. It builds on modeling frameworks previously developed in the context of continuous simulation of rainfall fields^45^, ^46^, ^47^ and radar rainfall error fields.^48^ The proposed method is based on Sklar's theorem^49^ extended to conditional distributions,^37^ which allows the decomposition of a conditional joint distribution into marginal distributions and a copula, thus splitting the modeling of the marginal temporal dependence structure and joint spatial dependence under the hypothesis of separability of spatio‐temporal correlation function. This approach is well known in econometrics^38^, ^39^ but is less common in hydrometeorology.^50^, ^51^

Referring to the literature for thorough introductions to copula theory and applications,^52^, ^53^, ^54^, ^55^ we recall basic properties of conditional copulas. Copulas are multivariate distributions that allow for construction of joint distributions with arbitrary marginals using Sklar's theorem. Focusing on a generic *d*‐dimensional case, we can write $F_{X}\left(x_{1},\ldots ,x_{d}\right)=C\left(F_{X_{1}}\left(x_{1}\right),\ldots ,F_{X_{d}}\left(x_{d}\right)\right)$, where $X=\left\{X_{1},\ldots ,X_{d}\right\}$ is a vector of *d* generic random variables with marginal distributions $F_{X_{i}}$, for i=1,…,d, and *C* is their copula. Since the copula theory was originally developed for (time) independent and identically distributed (iid) multivariate random variables, it may not be appropriate to describe many real‐world problems, such as persistent hydroclimatic time series at multiple locations or multiple sequences of stock price values, for which this hypothesis is not realistic. However, the concept of conditional copulas^37^ allows us to overcome this limit. In more detail, time dependence can be modeled by conditioning $X_{t}$ on the previous observations $X_{t},\ldots ,X_{t-k}$ and other generic exogenous variables **W**. In the time series context, Sklar's theorem may be extended as follows:^37^, ^38^, ^56^

(2) $$\begin{array}{ccc} & & F_{X,t}\left(x_{1},\ldots ,x_{d}|A_{t}\right)\\ & & \;=C_{t}\left(F_{X_{1},t}\left(x_{1}|A_{t}\right),\ldots ,F_{X_{d},t}\left(x_{d}|A_{t}\right)|A_{t}\right), \end{array}$$

where $C_{t}$ is the copula at times *t*, and

(3) $$A_{t}=\sigma \left\{x_{t},\ldots ,x_{t-k},w\right\}$$

is the σ‐algebra generated by all past joint information up to time *t* provided by the sample $x_{t-1}=\left\{x_{1,t-1},\ldots ,x_{d,t-1}\right\},\cdots$, $x_{t-k}=\left\{x_{1,t-k},\ldots ,x_{d,t-k}\right\}$, and possible covariates $w=\left\{w_{1},\ldots ,w_{n}\right\}$. In order for $F_{X,t}$ to be a valid conditional joint distribution function, Sklar's theorem for conditional distributions requires that the conditioning set $A_{t}$ must be the same for both marginal distributions and the copula. However, when each variable depends on its own previous lags but not on the lags of any other variables, Equation (2) describes a valid conditional distribution.^57^ Since in the present context, the random variables **X** are the weekly maxima of daily average stream flow recorded at *d* different sites and $A_{t}$ denotes the at‐site information related to the past flow values, the previous hypothesis corresponds to assuming that the cross‐correlation (spatial correlation) can be studied independently of the autocorrelation (temporal correlation), namely, that the spatio‐temporal correlation function is separable (in space and time components). The conditional copula method is also referred to as the dynamic copula method when it involves the modeling of the time‐varying marginals and dependence structures.

The separability hypothesis allows for splitting the analysis and modeling of marginals and dependence structure as follows.^58^, ^59^, ^60^

First, the *d* time series of weekly maximum values are preliminarily standardized by normal quantile transformation (NQT) and deseasonalized. Then the deseasonalized series are modeled by suitable time series models. Based on previous analyses on the same data set,^16^ we used three different options: (i) autoregressive fractionally integrated moving average (ARFIMA) models that allow the reproduction of both short‐ and long range‐ (time) dependence, (ii) autoregressive moving average (ARMA) models including only short‐range dependence, and (iii) a benchmark iid Gaussian noise. These three models for temporal dependence allow for the assessment of the impact of temporal correlation on the basin‐wide aggregated proxy losses.

Second, exploiting the separability hypothesis, the residuals of the univariate time series models at different locations and time steps *t* are temporally independent copies of *d* spatially dependent random variables that can be modeled by a multivariate distribution. Among the available options, only few classes of copulas/multivariate distributions allow effective high‐dimensional extensions preserving the variability of the degree of association between different locations. These classes include the so‐called vine copulas,^61^, ^62^ meta‐elliptical copulas,^63^ and extensions of Gaussian and Student multivariate distributions such as skew‐normal and skew‐*t*,^64^, ^65^ and v‐copulas.^66^

Vine copulas allow for building dependence structures suitable for describing conditional relationships of random variables naturally organized according to a hierarchical (tree‐like) structure, such as flow records across a river network. Based on these properties, vine copulas have been applied in collective flood risk/loss assessment;^30^, ^31^ however, since this class requires the definition of d(d−1)/2 bivariate copulas, and the *d* variables can be combined to form up to d!/2 different *d*‐dimensional vine copulas,^67^ only simplified versions (accounting for a reduced number d−1 of possible mutual conditional relationships) were applied in large‐scale analyses. On the other hand, meta‐elliptical copulas and, more generally, multivariate distributions involving a correlation matrix (such as Gaussian and Student, and v‐copulas) describe all d(d−1)/2 mutual pairwise relationships, but do not account for explicit modeling of possible hierarchical structures. Nonetheless, such a class of copulas was also used in flood risk assessment along a river network.^25^

In this study, we adopt the latter approach based on meta‐elliptical models, thus allowing for the description of all the mutual pairwise correlation values. It should be noted that the selection of maximum values within the one‐week time window of the hours clause tends to cancel out the effect of delays related to the flood routing across the river networks, thus making nonhierarchical models a suitable option. To assess the effect of the spatial correlation structure on the aggregated risk, three different dependence structures were considered: (i) full independence corresponding to the so‐called product copula, which is used as a benchmark; (ii) a *d*‐dimensional Gaussian copula:

(4) $$C_{G}\left(u;\Sigma \right)=\Phi_{\Sigma }^{d}\left(\Phi^{-1}\left(u_{1}\right),\ldots ,\Phi^{-1}\left(u_{d}\right)\right),$$

where $\Phi_{\Sigma }^{d}$ is a *d*‐dimensional Gaussian distribution with correlation matrix Σ, $u_{i}=F_{X_{i}}\left(x_{i}\right)$, and $F_{X_{i}}$ is the univariate distribution of $X_{i}$, for i=1,…,d; and (iii) a grouped *t*‐copula,^68^ which is a generalization of the classical Student *t*‐copula:

(5) $$C_{t}\left(u;\Sigma ,\nu \right)=t_{\Sigma ,\nu }^{d}\left(t_{\nu }^{-1}\left(u_{1}\right),\ldots ,t_{\nu }^{-1}\left(u_{d}\right)\right),$$

where $t_{\Sigma }^{d}$ denotes a centered multivariate Student distribution with correlation matrix Σ and univariate Student marginals $t_{\nu }$ with ν degrees of freedom. A grouped *t*‐copula arises as follows:^68^ let $Z∼\Phi_{\Sigma }^{d}$ be independent of a standard uniform random variable *U*, and $G_{\nu }$ the distribution function of $\sqrt{\nu /S}$, where $S∼\chi_{\nu }^{2}$. If we partition 1,…,d into *m* subsets of sizes $s_{1},\ldots ,s_{m}$, and define $R_{k}=G_{\nu }^{-1}\left(U\right)$, for k=1,…,m, therefore the vector

(6) $$Y={\left(R_{1}Z_{1},\ldots ,R_{1}Z_{s_{1}},R_{2}Z_{s_{1}+1},\ldots ,R_{2}Z_{s_{1}+s_{2}},\ldots ,R_{m}Z_{d}\right)}^{⊤}$$

follows a grouped *t*‐distribution, meaning that the first *s*_1_ components ${\left(Y_{1},\ldots ,Y_{s_{1}}\right)}^{⊤}$ follow an *s*_1_‐dimensional *t*‐distribution with ν_1_ degrees of freedom, and each of the *m* subsets ${\left(Y_{s_{1}+}\ldots +s_{k}+1,\ldots ,Y_{s_{1}+\ldots +s_{k+1}}\right)}^{⊤}$ is distributed as $s_{k+1}$‐dimensional *t*‐distribution with $\nu_{k+1}$ degrees of freedom. Therefore the vector

(7) $${\left(t_{\nu_{1}}\left(Y_{1}\right),\ldots ,t_{\nu_{1}}\left(Y_{s_{1}}\right),t_{\nu_{2}}\left(Y_{s_{1}+1}\right),\ldots ,t_{\nu_{2}}\left(Y_{s_{1}+s_{2}}\right),\ldots ,t_{\nu_{m}}\left(Y_{d}\right)\right)}^{⊤}$$

is a random variable from the grouped *t*‐copula, which offers therefore further flexibility by a set of $\nu_{k}$ parameters controlling the tail dependence and so the simultaneous occurrence of extreme events. The grouping procedure allows us to cluster together sets of time series according to some common behavior or property (e.g., membership in a given basin, basin similarities, or other factors). In the present study, time series are grouped according to the overall strength of association among the residuals of the AR(FI)MA models fitted on the deseasonalized series, under the assumption that the tails tend to cluster similarly to the body of the distribution. Of course, other options are possible, but this aspect is secondary in the present discussion. The combination of three marginal (temporal) dependence structures (independence (IND), ARMA, and ARFIMA) and three spatial dependence structures (independence (IND), Gaussian (GAU), and grouped *t*‐copula (GT)) results in nine model configurations (denoted as IND‐IND, IND‐GAU, IND‐GT, etc.) that are used to assess the impact of time persistence, spatial correlation, and their combination on the aggregated proxy losses (weighted areal hazard).

The modeling and simulation procedure can be summarized as follows:

(1) Each time series of stream flow weekly maxima is preprocessed by applying the NQT $z=\Phi^{-1}\left(F_{n}\left(q\right)\right)$, where $\Phi^{-1}$ denotes the inverse of the standard Gaussian cumulative distribution function and $F_{n}\left(q\right)=1/\left(T+1\right)\sum 1_{\left\{q_{t}\leq q\right\}}$ is the Weibull version of the empirical cumulative distribution function. These standardized series are therefore deseasonalized by subtracting the empirical average value for each calendar week $m_{i}$, i=1,…,52, from each observation $z_{t},i$, and then dividing by the calendar‐day standard deviation $s_{i}$, i=1,…,52. 69
(2) These deseasonalized time series are then modeled by AR(FI)MA models, which are used to model the at‐site information $A_{t}$, where $A_{t}=\sigma \left\{x_{t-1},\ldots ,x_{t-k},\varepsilon_{t-1},\ldots ,\varepsilon_{t-k}\right\}$, and $x_{t-j}$ and $\varepsilon_{t-j}$ denote the deseasonalized flow values and the AR(FI)MA residuals at times t−j, respectively. The term $\varepsilon_{t-j}$ corresponds with **w** in Equation (3). The optimal orders of the autoregressive and moving average components were selected by an automatic selection procedure based on the Akaike information criterion,^70^ which is known to be asymptotically equivalent to a leave‐one‐out cross‐validation procedure.
(3) The dependence structure of the temporally uncorrelated (but spatially correlated) residuals are therefore modeled by Gaussian copulas and grouped *t*‐copulas. For both copulas the required correlation matrix is estimated on a weekly basis (to account for seasonal fluctuations of the spatial correlation) by using the Spearman rank correlation coefficient $\rho_{\text{S}}$ and then the relationship between $\rho_{\text{S}}$ and the Pearson correlation coefficient $\rho_{\text{P}}=2\sin\left(\rho_{\text{S}}\right)/6$. This allows for a nonparametric estimation that is not affected by the possible failure of the hypothesis of Gaussian marginals and is effective if the spatial and/or temporal dependence structures are meta‐elliptical. The correlation matrices are finally corrected to guarantee the positive definiteness.^71^ For the grouped *t*‐copulas, eight groups were identified by a hierarchical clustering procedure based on the dissimilarity matrix of the AR(FI)MA residuals and Ward's minimum variance method. For each group the number of degrees of freedom is computed by the maximum likelihood method.
(4) To simulate space–time dependent flood hazard scenarios (over the stream gauge locations), the estimated (weekly) correlation matrices are used to simulate suitable *d*‐dimensional vectors following a multivariate Gaussian distribution and grouped *t*‐copula with Gaussian marginals (see Daul *et al*.^68^ for details on grouped *t*‐copula simulation). These spatially correlated vectors are temporally independent.
(5) The temporally independent sequences resulting from step (4) for each spatial location are used as spatially correlated innovations to feed at‐site AR(FI)MA models. This allows for the simulation of random sequences x that are both spatially and temporally correlated.
(6) Finally, the seasonal component is reintroduced by applying the transformation $z_{t}=x_{t},i\cdot s_{i}+m_{i}$, i=1,…,52, and the resulting series are transformed into a sequence of annual nonexceedance probabilities by
  (8) pta=1−52(1−Φ(zt))=1−521−ptw if ptw>5152pta=0 if ptw≤5152,
  where $p^{\text{w}}$ denotes the weekly nonexceedance probability. This procedure removes simulated values corresponding to virtual flow values exceeded more than once a year, for which the annual exceedance probability $\left(1-p^{\text{a}}\right)$ is equal to unity. Hence, the resulting scenarios preserve only values of potential interest for design and management, discarding all the low virtual hazard values.

## 3. MODELING RESULTS

To assess the performance of the modeling framework in terms of aggregated proxy losses, the hazard sequences $p_{t}^{\text{a}}$ are multiplied by the standardized values of exposure and vulnerability according to Equation (1), as discussed in Section 2.1.. These proxy losses are then aggregated at the basin scale and analyzed on a monthly and annual basis. Model performance is evaluated by a set of indices: (i) magnitude of weekly and annual losses, (ii) weekly probability of observing a loss, (iii) number of simultaneous weekly and annual losses (i.e., number of basins experiencing losses simultaneously), (iv) pairwise spatial correlation of weekly and annual loss time series for each pair of basins in the study area, and (v) the probability of observing simultaneous weekly losses for each pair of basins in the study area. The first two indices quantify the performance of the at‐site (marginal) components of the model, while the other three measures allow for checking the effect of the spatial components in terms of magnitude and frequency of basin‐wide proxy losses.

**Figure 2:**
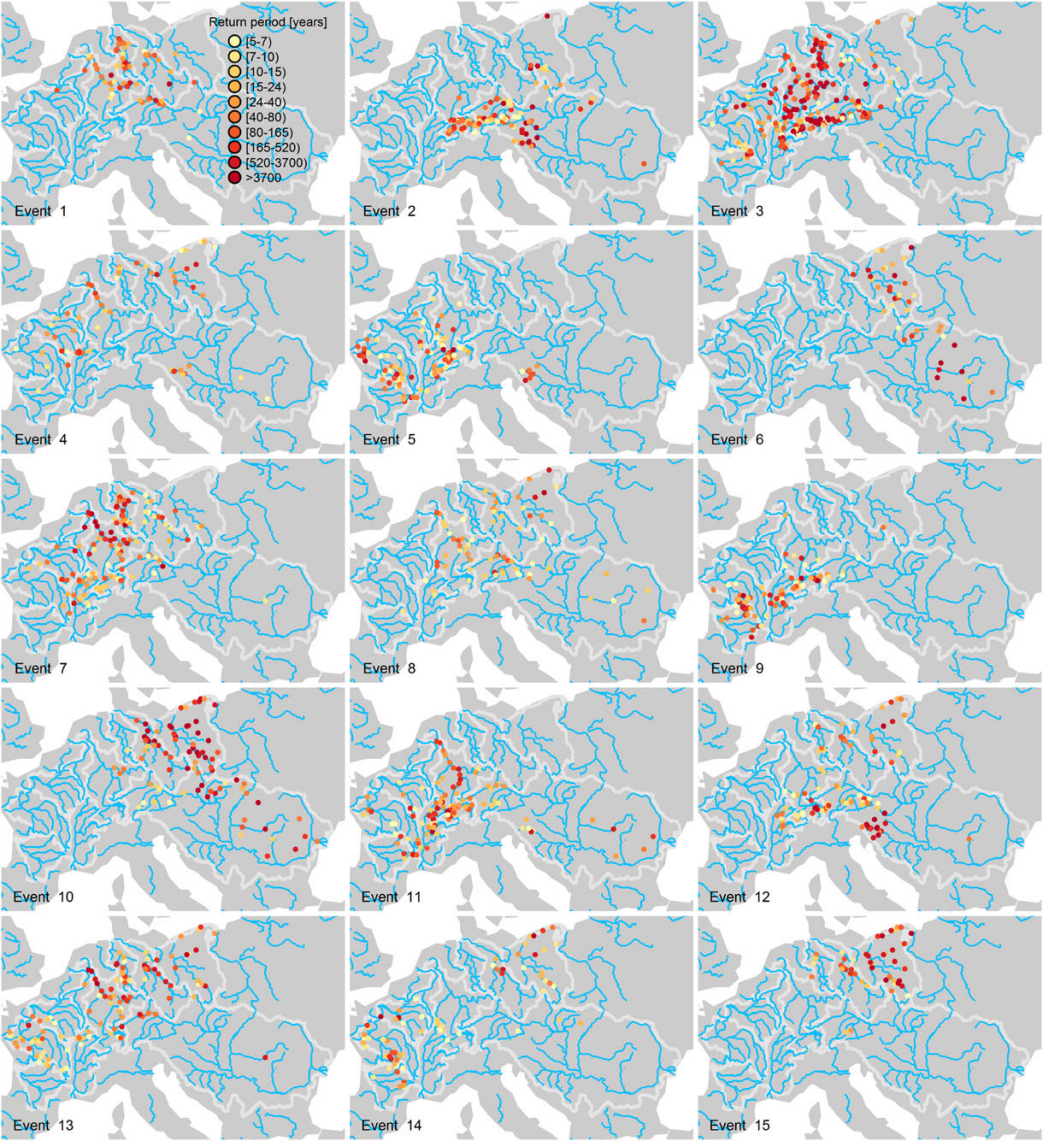
Fifteen event sets simulated by GT‐ARFIMA. Each panel shows “active” locations characterized by weekly maxima with return period equal to or larger than five years.

Before analyzing the model results in terms of performance indices, Fig. 2 shows some examples of events simulated by the model incorporating spatial dependence via grouped *t*‐copula and temporal dependence via ARFIMA models (GT‐ARFIMA). Each panel shows the flooding “active” locations where the at‐site discharge return period is equal to or larger than five years. These events show well‐defined spatial patterns involving, for instance, the lower Rhine, Elbe, and Weser (event 1), or the Alpine region (event 2), or the Rhine, Weser, and upper Danube (event 3), or the Elbe and Oder (event 15). Despite the purely statistical nature of the hazard model, it is able to reproduce physical properties such as the simultaneous (within one‐week window) flooding condition along the main stream of the Rhine (events 1, 3, 4, 7, 8, 11, 13), Weser (events 1, 3, 7, 10, 13), and Elbe (events 7, 8, 10, 13, 15). Physical coherence is less evident for the other rivers because of the limited number of stations available along the main stream as well as the relative disconnection between subcatchments feeding the main river,^31^ as is the case of the Danube, for instance.

**Figure 3:**
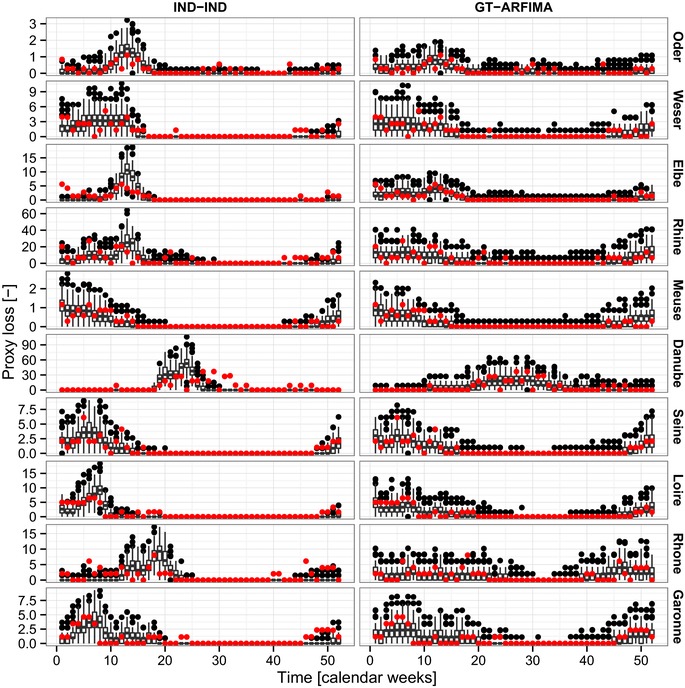
Observed and simulated weekly proxy losses at the basin scale for the two models IND‐IND (a) and GT‐ARFIMA (b). Red dots refer to the observed values, while simulated values are summarized by box plots in black (color visible in on‐line version).

Fig. 3 compares the observed weekly proxy losses aggregated over the 36 years with maximum data overlap (see Section 2.2.) and the values obtained by 277 samples of 36 years extracted from 10,000 simulated years for the simplest and most complex models in terms of spatio‐temporal structure: the first model is the simplest benchmark involving spatial and temporal independence (IND‐IND), while the second is GT‐ARFIMA. Since the focus is on weekly values, stream flow seasonality dominates the patterns of the proxy losses, but introducing spatial and temporal dependence structures results in increased variability (more evident in the low flow periods) and persistence (evident in the smoothed seasonality characterizing the high‐flow periods).

**Figure 4:**
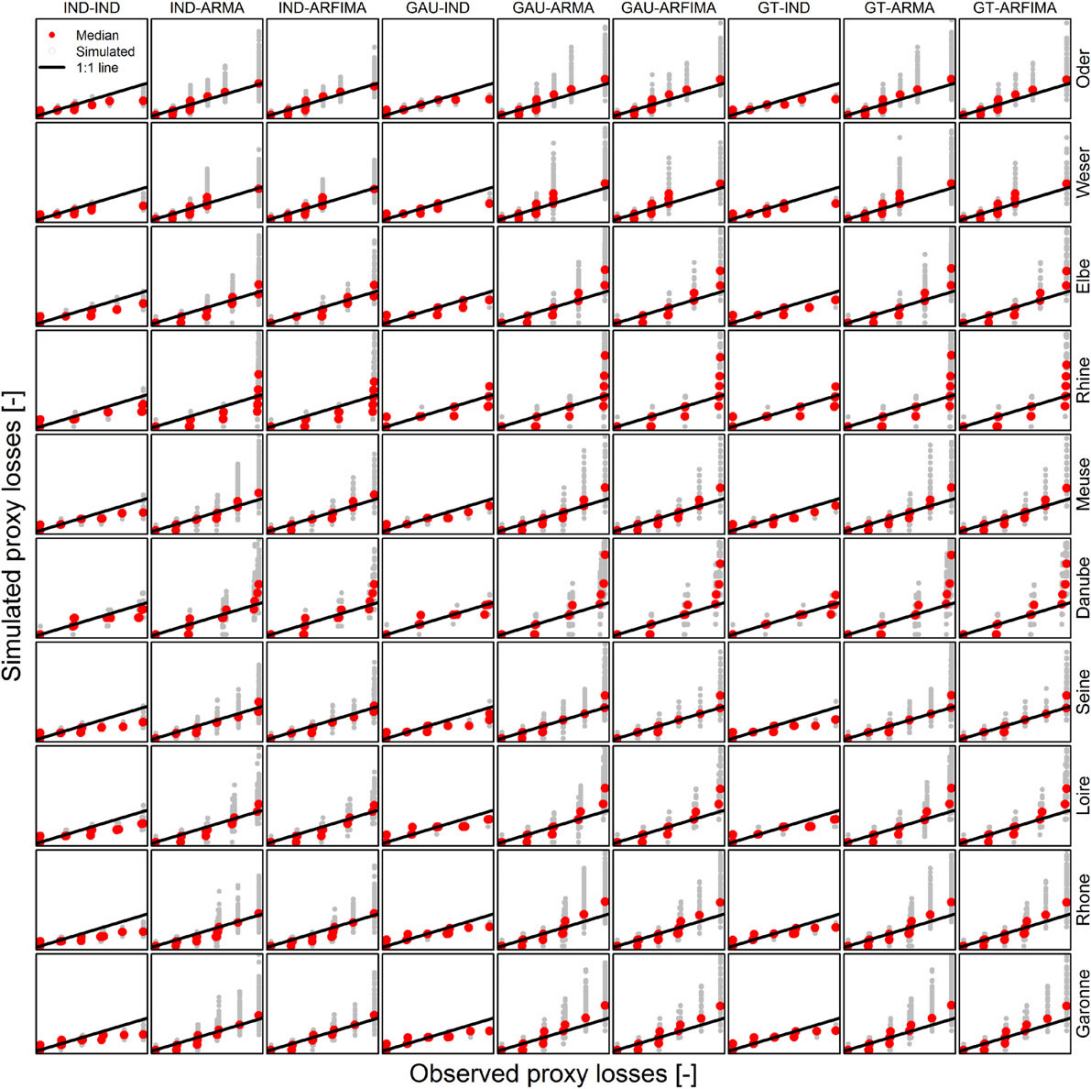
q–q plots of annual proxy losses. Range of simulated values and the median are shown.

This behavior generally holds true for the other model combinations as well; however, the separate effect of spatial and temporal dependence is more evident in the basin‐wide proxy losses aggregated at the annual time scale. Fig. 4 summarizes modeling results in terms of q–q plots for all the basins and model combinations. The models involving temporal independence (IND‐IND, GAU‐IND, GT‐IND) tend to underestimate both the median values (i.e., tend to be biased) and the interannual variability of the magnitude of the proxy losses. On the other hand, there is little difference between ARMA and ARFIMA results. This depends on the automatic selection procedure, which can yield ARMA configurations able to mimic long‐range dependence properties of ARFIMA models. Indeed, the main difference between these two alternatives is the smaller number of parameters of the fitted ARFIMA models compared to ARMA. In other words, the fractional differencing term is able to account for most of the temporal dependence, thus resulting in more parsimonious optimal models when compared with ARMA, which in turn require a larger number of parameters to describe the observed time series. Since the presence of long‐range dependence in stream flow time series was widely recognized in the literature,^72^, ^73^, ^74^, ^75^, ^76^ ARFIMA models seem to offer a coherent option, bearing in mind their limits.^77^

**Figure 5:**
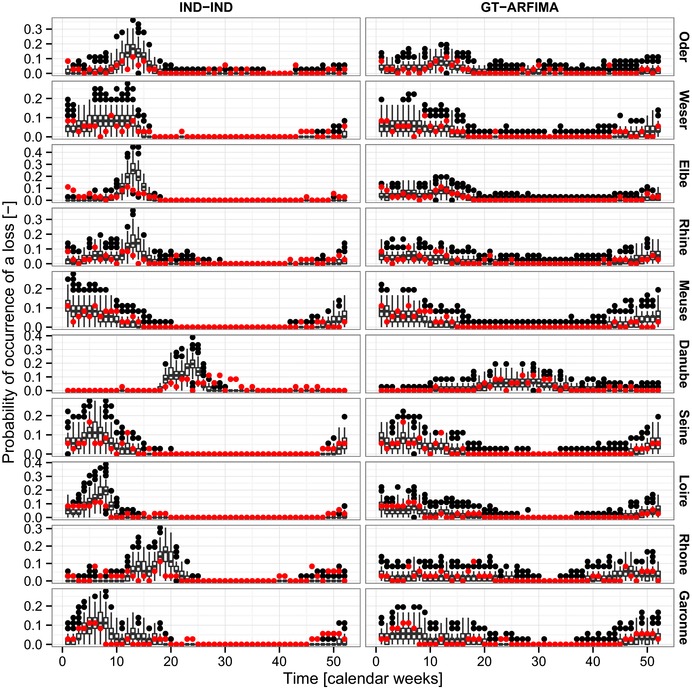
As for Fig. 3, but for the probability of observing a loss at the basin scale.

Fig. 5 shows the probabilities of observing a loss in each basin and calendar week for observed series, and IND‐IND and GT‐ARFIMA simulated sequences. As for the losses' magnitude, seasonality is the most evident feature, along with the higher variability and persistence characterizing the models involving temporal dependence structure.

**Figure 6:**
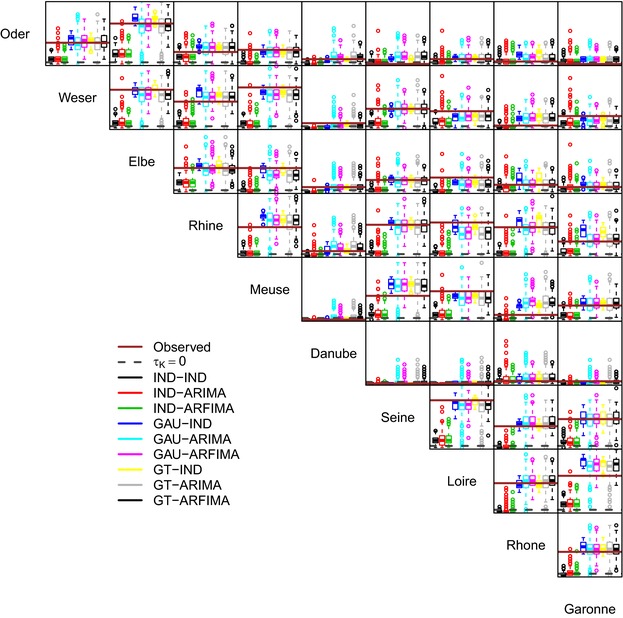
Pairwise (spatial) Kendall correlation values of weekly losses. Each panel shows the reference zero correlation, the observed value in the 36‐year time window discussed in the text, and the range of values corresponding to 277 36‐year‐long series simulated by the nine model configurations involving different structures/strength of the spatial and temporal dependence.

**Figure 7:**
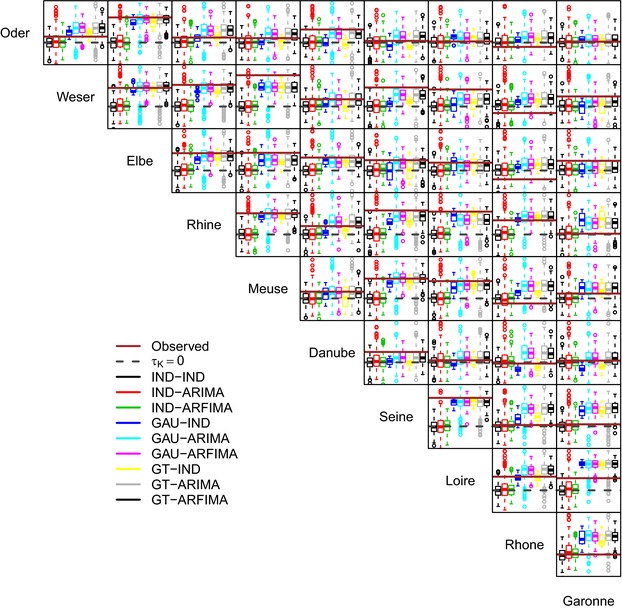
As for Fig. 6, but for annual proxy losses.

Fig. 6 shows the pairwise values of the Kendall correlation coefficient $\tau_{\text{K}}$ between the weekly proxy losses at the basin scale. It should be noted that the good reproduction of the correlation of basin‐wide losses is an indirect result of the spatial component of the model reproducing the spatial correlation among at‐site hazard time series. Therefore, even though some discrepancies are expected, the overall performance is rather satisfactory, with little difference between GAU and GT dependence structures. Performance deteriorates when losses are aggregated at the annual scale (Fig. 7). However, in 28 cases of 45, spatial dependence structures yield values closer to the observed than the IND setting. In a few cases, GAU and GT structures reproduce approximately the observed spatial independence, while in the remaining cases there is a tendency to overestimate the spatial correlation.

**Figure 8:**
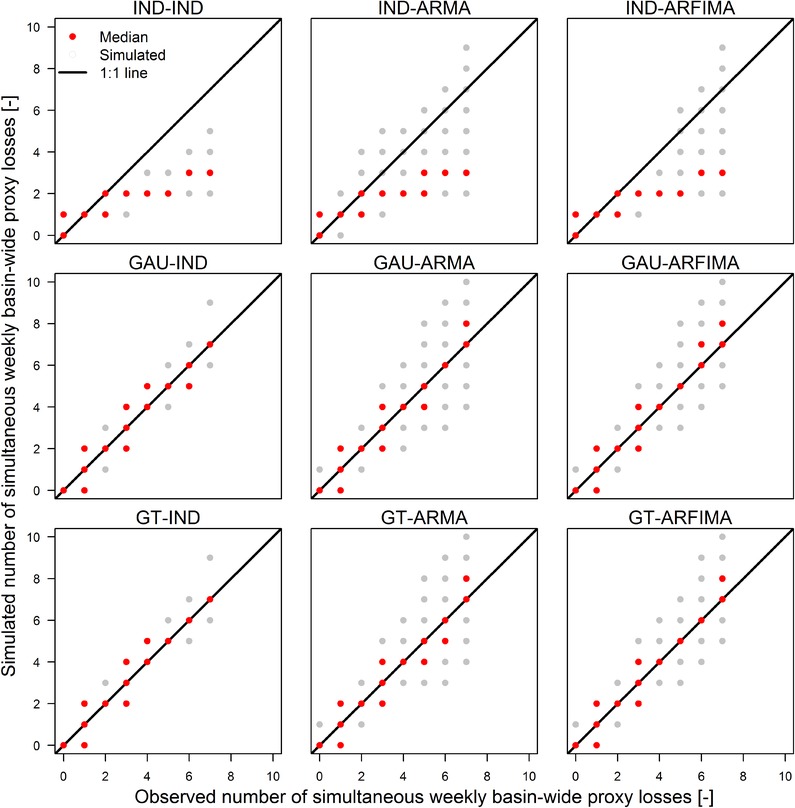
q–q plot of the number of basins exhibiting simultaneous proxy losses at the weekly time scale for the nine model configurations.

**Figure 9:**
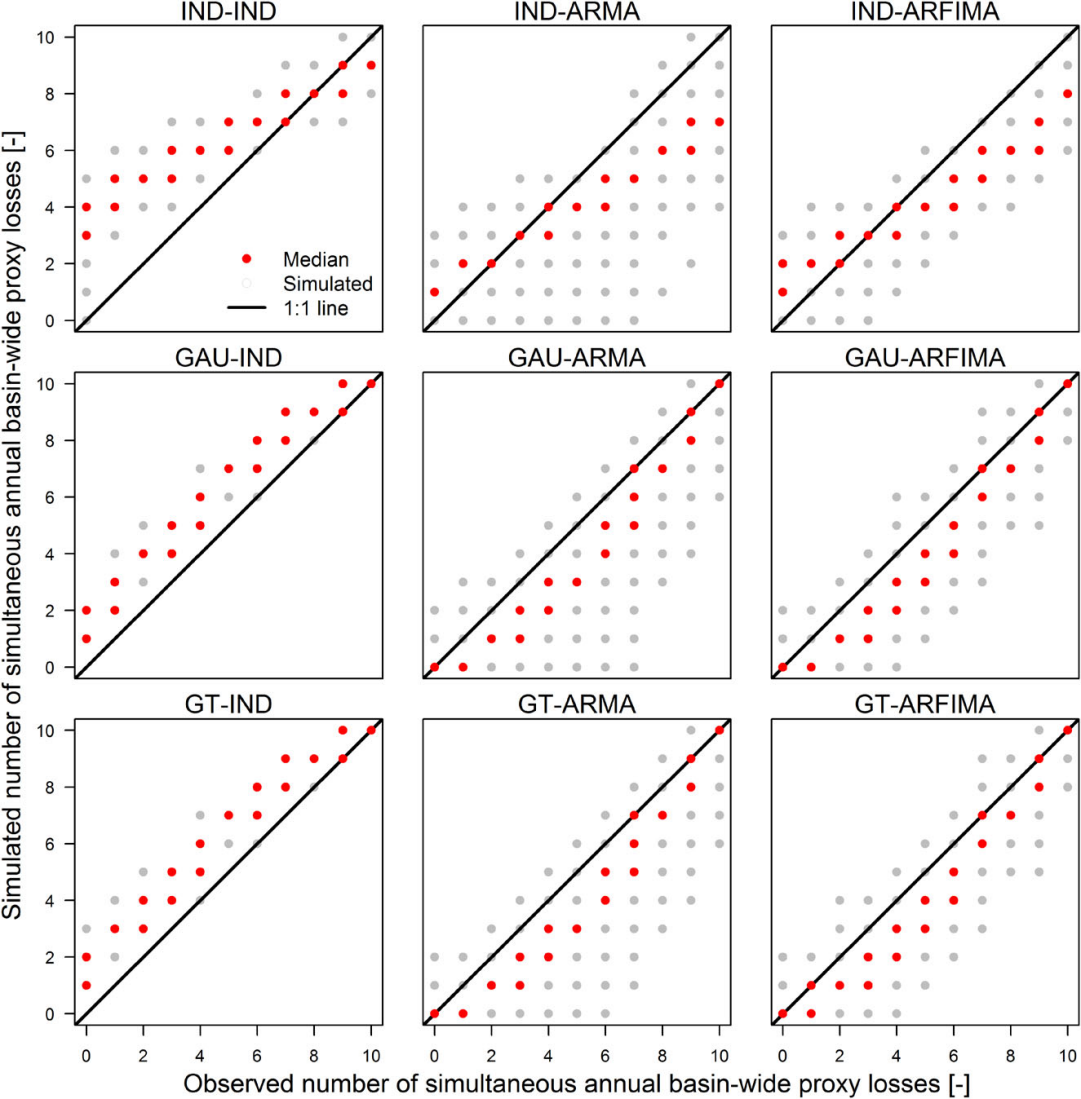
As for Fig. 8, but for proxy losses aggregated at the annual time scale.

An alternative assessment of the spatial dependence is given by the number of basins simultaneously affected by a basin‐wide loss the same week or year. Results for the nine model configurations are summarized in terms of q–q plots in Figs. 8 and 9 for weekly and annual scale, respectively. For weekly scale, models involving spatial independence (IND‐IND, IND‐ARMA, and IND‐ARFIMA) underestimate the number of simultaneous basins affected by proxy losses with exceedance probability lower than once per year (in average), while GAU and GT models correctly reproduce the observed behavior. Aggregation at the annual scale shows the effect of temporal and spatial structures. Models involving spatial independence show limited ability to describe the simultaneous occurrence of spatial losses, while GAU and GT models provide better results even though a residual bias is still present. This bias is positive when temporal dependence is not accounted for, while it is negative for models with AR(FI)MA components. However, ARFIMA structures yield values closer to the expected than ARMA, in terms of variability.

**Figure 10:**
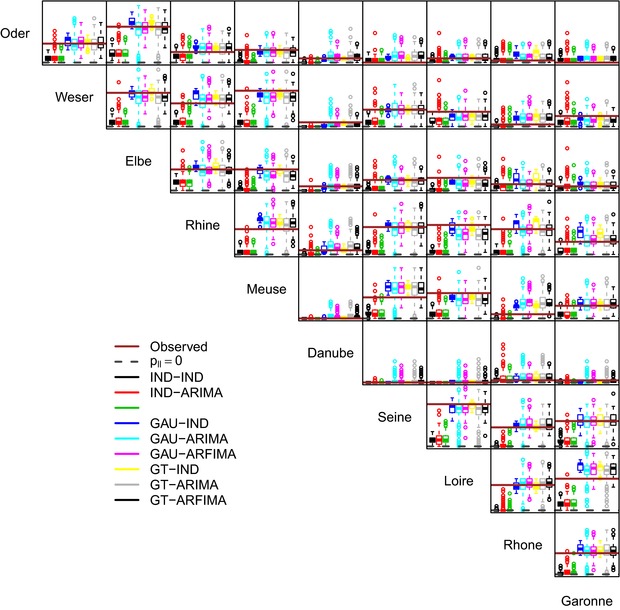
Pairwise probability of observing simultaneous weekly proxy losses in two basins in the study area.

We finally checked the framework capability of modeling the weekly probability $p_{ll}$ of observing simultaneous basin‐wide aggregated losses (with exceedance probability lower than once per year on average) in each pair of basins in the considered area. This information can be used for management strategies in order to optimize, for instance, (re)insurance policies accounting for simultaneous risk in different areas such as river basins or countries. Fig. 10 further confirms the advantages of introducing spatial dependence in a hazard model. Especially notable is the low probability of observing simultaneous losses in the Danube basin and the other European basins. This is mainly related to the different seasonal pattern characterizing the Danube basin, whose dynamics are driven by heterogeneous meteorological forcings acting both across the entire basin, which is much larger than the others, and in single subcatchments.^78^, ^79^, ^80^

## 4. CONCLUSIONS

Flood hazard is a fundamental component of design and management procedures for flood risk assessment. Flood hazard is usually quantified by flood frequency analyses based on extreme values such as annual maximum values or values over a high threshold. However, these approaches often rely on a limited number of data. In order to exploit the information contained in all the available data, we proposed a continuous simulation approach incorporating both spatial and temporal dependence. The proposed methodology is general and balances between a more efficient use of available data, operational aims (e.g., hours clause policy), and a relatively simple model architecture. In particular, we merge spatial and temporal dependence structures under the hypothesis of separability, which makes the conditional copula widely applicable to real‐world problems.

Using a large data set of daily stream flow time series recorded in the main river basins across central and eastern Europe, we assessed the performance of the model in terms of proxy losses aggregated at the basin spatial scale, and weekly and annual time scales. Proxy losses are defined as the product of hazard scenarios (observed or simulated), and vulnerability and exposure, which in turn are approximated by simple socioeconomic proxy indices easily retrievable from public databases. Such proxy losses can also be seen as weighted hazard values useful to quantify the areal (e.g., basin‐wide or state‐wide) hazard accounting for the effect of the local hazard magnitude in a simplified but effective fashion.

The proposed framework allows for the coupling of a variety of spatial and temporal dependence structures. In this respect, the choice of the optimal model structure (spatial and temporal modules) should be based on the reproduction of a suitable set of statistics of interest (such as spatially and temporally aggregated measures) rather than some standard indices involving the same quantities used in the fitting stage. The use of simple model benchmarks for the sake of comparison is also recommended along with model structures of intermediate complexity in order to evaluate the impact of the single components. From this point of view, the modular structure resulting from conditional copulas and the corresponding simple sampling algorithms allow for the relatively fast simulation of large samples from different model structures to be used for a post hoc validation.

From an operational point of view, based on the empirical results reported in this study as well as previous preliminary analyses on the same data set^16^ and conceptual remarks concerning the parsimony principle,^77^ models involving long‐range dependence, such as ARFIMA or generalized Hurst‐Kolmogorov,^81^ are suggested to describe the at‐site (marginal) stream flow dynamics. In fact, one or two parameters controlling short‐range dependence and one parameter describing long‐range dependence are often sufficient to summarize the key linear properties of stream flow signals. In this respect, fitting models not including a long‐range dependence component (e.g., ARMA) usually results in a larger number of parameters attempting to mimic long‐range dependence. As far as the spatial dependence structure is concerned, spatial independence is surely an oversimplified assumption overlooking river network connections and interbasin dependence, while a simple Gaussian copula is a more realistic option embedding observed pairwise correlation between stream gauges. On the other hand, the additional parameters (degrees of freedom) of grouped *t*‐copulas allow the control of upper tail dependence, that is, the joint occurrence of extreme values as well as the tuning of this property for subsets of stream flow series clustering together according to some physical criterion such as membership in a given drainage basin or subcatchment or in the main stream of a river network. Moreover, grouped *t*‐copulas yield standard *t*‐copulas when the number of groups is equal to one, and they tend to converge to Gaussian copulas for large values of degrees of freedom. Therefore, *t*‐copulas introduce useful flexibility with a moderate number of additional parameters and include other simpler models as special cases, making them a suitable compromise between too simple competitors and more complex (highly parameterized) models.

Empirical results show the importance of accounting for spatial and temporal dependence in hazard modeling to reproduce the simultaneous occurrence of extreme events, interannual variability, and persistence. Continuous simulation also allows for a more accurate simulation of spatial hazard events by identifying and reproducing coherent spatial patterns along the calendar year. This information can be used to refine flood management strategies identifying the areas simultaneously prone to flood hazard, and quantifying the corresponding probability to have joint regional losses. Finally, it should be noted that the modeling framework can easily be adapted and improved by introducing different spatial components (i.e., hierarchical dependence structures), and temporal modules (e.g., nonlinear temporal models) in order to refine the analysis or adapt it to other geophysical variables and hazards.

## Footnotes

1 In number of inhabitant per ${\text{k}m}^{2}$.

2 In purchasing power standards (PPS) per inhabitant.

3 The NUTS classification (Nomenclature of Territorial Units for Statistics) is a hierarchical system for dividing up the economic territory of the European Union for statistics collection, socioeconomic analyses, and framing regional policies. It comprises 1,342 regions at the finer spatial resolution.

4
http://ec.europa.eu/eurostat/statistics-explained/index.php/Population_statistics_at_regional_level; http://ec.europa.eu/eurostat/statistics-explained/index.php/Archive:GDP_and_household_accounts_at_regional_level

5
http://www.eea.europa.eu/data-and-maps/data/dominant-land-cover-types-2000-1
